# Analysis of thoracoscopic pulmonary wedge resection, thoracoscopic lobectomy and open lobectomy in patients with lung cancer

**DOI:** 10.4314/ahs.v25i2.16

**Published:** 2025-06

**Authors:** Feibao Jiang, Deguang Pan, Yanjun Qiu

**Affiliations:** 1 Surgical department Ward one, People's Hospital of Changshan County, Quzhou, China; 2 Department of Cardiothoracic Surgery, Chun'an County First People's Hospital, Hangzhou, China; 3 Department of Cardiothoracic Surgery, The Quzhou Affiliated Hospital of Wenzhou Medical University, Quzhou People's Hospital, Quzhou, China

**Keywords:** Thoracoscopic pulmonary wedge resection, thoracoscopic lobectomy, open lobectomy, lung cancer, surgery

## Abstract

**Background:**

To analyze the value of thoracoscopic lung wedge resection, thoracoscopic lobectomy and open lobectomy in patients with lung cancer.

**Methodology:**

Ninety-six patients with lung cancer were divided into three groups: thoracoscopic lung wedge resection (F1 group), thoracoscopic lobectomy (F2 group), and open lobectomy (F3 group). The study assessed the patients' general condition, pulmonary function, oxidative stress indicators, quality of life (EORTC QLQ-C30), and occurrence of postoperative complications, including pulmonary infections.

**Results:**

The F1 and F2 groups outperformed the F3 group in most surgical indicators, except for lymph node count. F1 group had better results than the F2 group. Pulmonary function was better in the F1 group postoperatively compared to F2 and F3 groups. F1 and F2 groups exhibited lower levels of oxidative stress indicators, while having higher levels of SOD than the F3 group. At 3 and 6 months' post-surgery, F1 group had lower EORTC QLQ-C30 scores than F2 and F3 groups. The incidence of complications, including lung infections within 6 months after surgery, was lower in F1 and F2 groups than the F3 group.

**Conclusion:**

Thoracoscopic lung wedge resection can reduce surgical trauma and oxidative stress, alleviate lung function damage and postoperative complications, and improve the level of quality of life.

## Introduction

Surgery is currently the most effective clinical treatment option for lung cancer^1^. Minimally invasive surgery has now become an important concept in thoracic surgery, and for patients with lung cancer, the key challenge is how to reduce trauma^2^. Traditional open lobectomy is more invasive in the treatment of this disease, while video-assisted thoracic surgery (VATS) is especially suitable for elderly patients with poor body tolerance. With the in-depth study of thoracoscopic techniques, more thoracoscopic procedures are gradually applied in the treatment of lung cancer, but their efficacy is also somewhat different 3, 4. In view of this, this paper mainly analyses the therapeutic effects of 2 types of thoracoscopic procedures and traditional procedures.

## Patients and methods

*Patients*. Ninety-six patients with lung cancer admitted to our hospital from September 2018 to September 2020 were retrospectively selected. The patients met the relevant diagnostic criteria^5^, and the inclusion criteria were as follows: (1) patients had clear indications for surgery by imaging such as Computed Tomography (CT) and chest radiographs; (2) patients had no organ dysfunction such as heart, liver or kidney, and no history of radiotherapy or chest adhesions within the last 1 week; (3) patients had TNM stage T1-2N0M0 and signed the informed consent for surgery; (4) the surgical option was selected based on the patient's condition, and for lung poor functional tolerance and for those who cannot easily undergo lobectomy under the principle of ensuring radical treatment, lung wedge resection was chosen. The exclusion criteria were as follows: (1) patients with severe psychiatric or cardiovascular disease or serious organic lesions; (2) patients with previous pacemaker placement or coronary stenting or distant lymph node metastases. The three groups were grouped into F1, F2 and F3 according to the surgical procedure. 32 cases were identified in all three groups, and there was no difference in their general data (P>0.05), See Table 1.

**Table 1:** Comparison of general data

Group	Genders		Age (years)	Pathological type		
	
	Male	Female		Adenocarcinoma	Squamous cell carcinoma	Adenosquamous carcinoma
Group F1 (n = 32)	18 (56.25)	14 (43.75)	53.29 ± 5.46	12 (37.50)	11 (34.38)	9 (28.12)
Group F2 (n = 32)	17 (53.12)	15 (46.88)	53.37 ± 5.49	10 (31.25)	12 (37.50)	10 (31.25)
F3 group (n = 32)	19 (59.38)	13 (40.62)	53.11 ± 5.48	11 (34.38)	12 (37.50)	9 (28.12)
Statistic	0.254		0.019	0.310		
P-value	0.881		0.981	0.989		

*Surgical methods*. All three groups were given the same specification of nursing interventions and were treated with appropriate pharmacological support according to their condition. In group F1, a thoracoscopic lung wedge resection was performed with a thoracoscopic viewing port and a 3-5 cm operating port, and the operation was started under monitor guidance. In group F2, a thoracoscopic lobectomy was performed: the main operating hole and the secondary operating hole were made, the lobe was removed and the lymph nodes were completely cleared. in group F3, an open lobectomy was performed: a posterior lateral thoracotomy was made and the lobe was routinely removed and the mediastinal lymph nodes were completely cleared by blunt separation into the chest layer by layer.

*Outcome measures*. The operation conditions were recorded. The pulmonary function of the three groups was measured by spirometer before surgery and 3 months after surgery, including FVC, FEV1%, PEF. The levels of cortisol (Cor) and endothelin-1 (ET-1) were measured by enzyme-linked immunosorbent assay kit, the content of malondialdehyde (MDA) was measured by TBA kit, and the level of superoxide dismutase (SOD) was measured by WST-1 kit; (4) The quality of life of the three groups was assessed by EORTC QLQ-C30 scale before surgery, 3 months after surgery, and 6 months after surgery, with a total of 30 items in this table, of which items 29 and 30 were forward scores, divided into seven grades, 1 to 7 points, and other items were divided into 4 grades, reverse scores, 1 to 4 points, full score 126 points, and the worse the quality of life; (5) Complications were recorded.

Statistical analysis. Statistical Product and Service Solutions (SPSS) 23.0 (IBM, Armonk, NY, USA) was applied for statistical analysis. Independent sample t-test was used for comparison between groups for measurement data obeying normal distribution, and independent sample t-test was used for comparison within groups, all expressed as (x̅±s). Count data were tested by *χ*^2^ and expressed as rate (%), P<0.05 indicates statistical difference.

## Results

Comparison of general surgical conditions among the three groups. There were significant differences among the three groups except for the number of lymph nodes (P < 0.05) (as shown in Table 2).

**Table 2:** Comparison of general surgical conditions among three groups

Group	Intraoperative blood loss (ml)	Surgical Incision (cm)	Operative Time (min)	Total Drainage (L)	Number of lymph nodes scanned (unit)
Group F1 (n = 32)	94.15 ± 9.87	6.53 ± 0.71	118.64 ± 12.34	1.34 ± 0.15	15.68 ± 1.64
Group F2 (n = 32)	114.52 ± 12.37 *	6.49 ± 0.82	145.68 ± 16.24 *	1.50 ± 0.16 *	16.10 ± 1.72
F3 group (n = 32)	165.45 ± 17.18#	18.41 ± 1.96#	171.89 ± 18.12#	1.68 ± 0.17 #	15.94 ± 1.86
F value	244.978	873.403	94.384	37.236	0.489
P-value	< 0.001	< 0.001	< 0.001	< 0.001	0.614

Note: Compared with group F1, P < 0.05; compared with group F2, P < 0.05

Comparison of pulmonary function before and after operation among the three groups. FVC, FEV1%, and PEF at 3 months after surgery in the three groups were lower than those before surgery in the same group, but FVC, FEV1%, and PEF after surgery in group F1 were higher than those in groups F2 and F3, and there were also significant differences in the above parameters between groups F2 and F3 (P < 0.05) (as shown in Table 3).

**Table 3:** Comparison of pulmonary function

Group	FVC (L)		FEV1% (%)		PEF (L/s)	

	Pre-op	3 months after operation	Pre-op	3 months after operation	Pre-op	3 months after operation
Group F1 (n = 32)	2.96 ± 0.31	1.64 ± 0.17	81.40 ± 8.26	70.15 ± 7.63	5.14 ± 0.52	4.92 ± 0.51
Group F2 (n = 32)	2.95 ± 0.34	1.32 ± 0.14 *	80.44 ± 8.31	64.27 ± 6.38 *	5.18 ± 0.53	3.74 ± 0.38*
F3 group (n = 32)	2.97 ± 0.28	1.07 ± 0.13#	81.38 ± 8.28	58.41 ± 5.96#	5.15 ± 0.50	2.86 ± 0.34 #
F value	0.034	123.694	0.145	25.398	0.054	203.567
P value	0.921	< 0.001	0.880	< 0.001	0.942	< 0.001

Note: Compared with group F1, P < 0.05; compared with group F2, P < 0.05.

Comparison of oxidative stress indicators among the three groups. After surgery, the levels of COR, MDA, and ET-1 in groups F1 and F2 were lower than those in group F3, while SOD was higher than that in group F3 (P < 0.05), the levels of COR, MDA, and ET-1 in group F1 were lower than those in group F2, and SOD was higher than that in group F3 (P < 0.05) (as shown in Table 4).

**Table 4:** Comparison of oxidative stress indicators among the three groups

Group	Cor (ng/mL)		MDA (nmol/mL)		ET-1 (pg/mL)		SOD (U/mL)	

	Pre-op	3 months after operation	Pre-op	3 months after operation	Pre-op	3 months after operation	Pre-op	3 months after operation
Group F1 (n = 32)	201.44 ± 20.89	143.25 ± 15.18	10.15 ± 1.23	7.54 ± 0.78	87.25 ± 8.86	65.43 ± 6.67	56.02 ± 6.45	74.26 ± 7.51
Group F2 (n = 32)	202.53 ± 20.76	162.33 ± 16.54 *	10.21 ± 1.18	8.05 ± 0.86*	87.33 ± 8.81	70.12 ± 7.18 *	56.13 ± 6.34	65.44 ± 6.67 *
F3 group (n = 32)	201.39 ± 21.43	187.34 ± 18.57#	10.13 ± 1.12	8.69 ± 0.94 #	87.39 ± 8.74	78.23 ± 7.96#	55.27 ± 6.25	56.19 ± 5.72 #
F value	0.030	55.293	0.040	14.284	0.002	25.255	0.174	58.665
P-value	0.970	< 0.001	0.961	< 0.001	0.998	< 0.001	0.841	< 0.001

Comparison of quality of life before and after surgery among the three groups. The EORTC QLQ-C30 scores of the three groups after surgery were lower than those before surgery, and the EORTC QLQ-C30 scores of group F1 were the lowest at each time point after surgery (P < 0.05) (as shown in Table 5).

**Table 5:** Comparison of quality of life

Group	Pre-op	3 months after operation	6 months after operation	F value	P-value
Group F1 (n = 32)	75.14 ± 7.69	48.15 ± 5.23	20.16 ± 2.78	794.924	< 0.001
Group F2 (n = 32)	76.15 ± 7.83 *	55.68 ± 5.34*	34.85 ± 3.97 *	400.227	< 0.001
F3 group (n = 32)	75.67 ± 7.96#	63.15 ± 6.47#	42.15 ± 4.63#	224.459	< 0.001
F value	0.138	57.037	276.694		
P-value	0.795	< 0.001	< 0.001		

Note: Compared with group F1, P < 0.05; compared with group F2, P < 0.05.

Comparison of the incidence rate of postoperative complications. The incidence rate of complications such as pulmonary infection in group F1 and F2 was lower than that in group F3 at 6 months after operation (P < 0.05). There was no significant difference in the incidence rate of complications between group F1 and F2 (P > 0.05) (as shown in Table 6).

**Table 6:** Comparison of complications n (%)

Group	Number of lung infections	Pneumonia cases	Number of atelectasis	Number of cases of empyema	Total Occurrence
Group F1 (n = 32)	0	0	1	0	1 (3.12) *
Group F2 (n = 32)	1	0	0	1	2 (6.25) *
F3 group (n = 32)	2	2	1	3	8 (25.00)
X^2^					8.830
P-value					0.012

Note: Compared with F3 group, P < 0.05.

## Discussion

Lung cancer is a common malignant tumor and has become the leading cause of death from malignant tumors in China, seriously threatening the lives of our residents^6^. Thoracoscopic techniques for the treatment of lung cancer have the advantages of good safety, aesthetic incision and few postoperative complications, and high tolerance for follow-up treatment with postoperative radiotherapy, but the impact on patients' lung function has yet to be confirmed by large sample size studies^7^, ^8^. At present, thoracoscopic pneumonectomy in China is mainly based on lobectomy and wedgeesection, while thoracoscopic anatomical segmental lung resection has been carried out, but strict indications are needed, of which wedge resection is particularly suitable for those who cannot tolerate lung function, and its surgical curve is also worth continuous learning^9^,^10^.

The current finding was that thoracoscopic lung wedge resection has the advantages of less trauma, lower drainage and shorter operative time. This may be due to the fact that traditional open lobectomy treatment requires severance of the chest wall muscles and ribs, excessive pulling of the spreader can lead to damage to the chest wall, greater intraoperative trauma and pressure compression of the lung making the operation more difficult. In contrast, thoracoscopic surgery was widely used to remove the diseased lung lobes of lung cancer patients with its features of less surgical trauma, less postoperative pain and faster recovery^11^, ^12^.

In terms of lung function, this study showed that FVC, FEV1% and PEF were all lower than those before surgery in the same group at 3 months after surgery in all three groups, but FVC, FEV1% and PEF were higher in group F1 than in groups F2 and F3 after surgery, and there was also a significant difference in the comparison of the above indicators between groups F2 and F3, indicating that all three types of surgery may affect lung function in lung cancer patients, but thoracoscopic surgery has less damage to patients' lungs, especially thoracoscopic. However, thoracoscopic lung wedge resection can significantly reduce the damage to lung tissue, which may be related to the extent of lung tissue resection selected, and thoracoscopic lobectomy preserves lung tissue to a certain extent, while thoracoscopic lung wedge resection can preserve segmental bronchi and normal lung tissue as much as possible, while the remaining lung expansion after thoracoscopic lobectomy plays a compensatory effect but still cannot compensate for the damage to lung tissue itself on its function. This is similar to the findings of other study^13^.

Thoracoscopic surgery trauma itself and postoperative incisional pain are strong stressors that can lead to the activation of oxidative stress in the body, where Cor is an important stress hormone that regulates substance metabolism. MDA and ET-1 are products of oxygen free radical attack on cells, and in addition a variety of antioxidant substances such as SOD are secreted in the body after oxidative stress^14^. In the current study, the levels of Cor, MDA and ET-1 in the F1 and F2 groups were lower than those in the F3 group, while SOD was higher than that in the F3 group, and the indicators in the F1 group were better than those in the F2 group, indicating that the postoperative oxidative stress caused by thoracoscopic lung wedge resection was the least severe.

In the study, it was also found that the F1 group had the best quality of life at 3 and 6 months after surgery, indicating that lung cancer patients benefited more from undergoing thoracoscopic lung wedge resection than thoracoscopic lobectomy and open abdominal lobectomy, and that thoracoscopic lung wedge resection was less invasive and had less injury to lung function, thus helping to improve the quality of life after surgery^15^, ^16^. This study has some limitations. For example, this is a retrospective study, thus the grouping is performed according to the surgical procedure rather than randomization. Additionally, this is a single center study with a small sample size. Given all above, the findings in the present study should be further validated by future studies.

## Conclusions

Thoracoscopic lung wedge resection and thoracoscopic lobectomy for lung cancer are less invasive and can better reduce complications such as lung function damage and lung infection, and improve the quality of life after surgery. For those who cannot tolerate lung function, thoracoscopic lung wedge resection has obvious advantages and is worth promoting in clinical practice.

## Data Availability

The datasets used and analyzed during the current study are available from the corresponding author on reasonable request.
